# Dual view deep learning for enhanced breast cancer screening using mammography

**DOI:** 10.1038/s41598-023-50797-8

**Published:** 2024-02-15

**Authors:** Samuel Rahimeto Kebede, Fraol Gelana Waldamichael, Taye Girma Debelee, Muluberhan Aleme, Wubalem Bedane, Bethelhem Mezgebu, Zelalem Chimdesa Merga

**Affiliations:** 1 Research Development Cluster, Ethiopian Artificial Intelligence Institute, Addis Ababa, 40782 Ethiopia; 2 College of Electrical and Mechanical Engineering, Addis Ababa Science and Technology University, Addis Ababa, 120611 Ethiopia; 3 College of Engineering, Debre Berhan University, Debre Berhan, Ethiopia; 4 Radiology, St. Pauli Millenium Medical College, Addis Ababa, Ethiopia; 5 Department of Surgery, Zewditu Memorial Hospital, Addis Ababa, Ethiopia; 6 Radiology, Pioneer Diagnostic Center, Addis Ababa, Ethiopia

**Keywords:** Breast cancer, Computer science, Biomedical engineering

## Abstract

Breast cancer has the highest incidence rate among women in Ethiopia compared to other types of cancer. Unfortunately, many cases are detected at a stage where a cure is delayed or not possible. To address this issue, mammography-based screening is widely accepted as an effective technique for early detection. However, the interpretation of mammography images requires experienced radiologists in breast imaging, a resource that is limited in Ethiopia. In this research, we have developed a model to assist radiologists in mass screening for breast abnormalities and prioritizing patients. Our approach combines an ensemble of EfficientNet-based classifiers with YOLOv5, a suspicious mass detection method, to identify abnormalities. The inclusion of YOLOv5 detection is crucial in providing explanations for classifier predictions and improving sensitivity, particularly when the classifier fails to detect abnormalities. To further enhance the screening process, we have also incorporated an abnormality detection model. The classifier model achieves an F1-score of 0.87 and a sensitivity of 0.82. With the addition of suspicious mass detection, sensitivity increases to 0.89, albeit at the expense of a slightly lower F1-score of 0.79.

## Introduction

Breast cancer has the highest incidence rate of 40.6 per 100,000 population among all cancers in Ethiopia in 2020^1^. Due to the lack of therapy and late diagnosis, the mortality rates from breast cancer in developing countries like Ethiopia are much higher^2^. In most of the developed world, more than 70% of breast cancer patients are diagnosed when the cancer is at its earlier stages I and II. However, only 20-50% of patients in the majority of low- and middle-income countries were diagnosed at early stages^3^. A study conducted in northern Ethiopia^4^ shows that about 85% of the cases diagnosed in Ethiopia were at an advanced stage III and IV.

A study cited in^5^ found that the 5-year survival rate for breast cancer detected at stage I, II, III, or IV is 98%, 93%, 63%, and 31%, respectively. This suggests that early detection is critical for improving the chances of survival. For early detection, women (especially those whose age is greater than 40) must perform breast self-exams, regular clinical breast exams, and mammograms^6^. Screening through mammography is one of the most effective and affordable methods for early detection of breast mass^7,8^.

Radiologists meticulously analyze mammography images and document their observations on any detected abnormalities, utilizing the Breast Imaging Reporting and Data System (BI-RADS) due to its user-friendly nature and provision of management guidance^9^. Nevertheless, to cultivate a screening culture among women, mass screening needs to be promoted; however, the limited number of radiologists makes this approach impractical in developing countries like Ethiopia. Therefore, the development of an AI model with high sensitivity in detecting breast abnormalities would assist radiologists in prioritizing cases and improving diagnostic accuracy. This paper presents an ensemble of classification and object detection algorithms aimed at identifying breast abnormalities, emphasizing the importance of early-stage detection. Currently, most cases are diagnosed at an advanced stage, underscoring the life-saving potential of early detection and making it a paramount responsibility for radiologists.

Machine learning and deep learning methods have found extensive applications in various domains, including disease detection and classification^10–17^. In the context of breast cancer detection and classification, classical machine learning methods have been commonly employed^18,19^. However, these classical methods require robust feature engineering to extract relevant features from a smaller dataset. Nonetheless, manual feature extraction techniques often fail to capture all the necessary features, resulting in the inclusion of irrelevant ones. In contrast, convolutional neural network (CNN) models have shown promising performance for breast cancer classification by automatically generating high-quality feature maps when efficiently trained^8^. These CNN-based models demonstrate encouraging results in distinguishing normal and abnormal mammograms^20,21^. Nevertheless, full image classification alone may be challenging to explain to radiologists and might not provide information on the precise location of abnormalities. To address these limitations, machine learning-based breast cancer detection and classification methods offer improved explanations and increased trustworthiness. Various techniques have been proposed for detecting abnormal regions in mammograms, including both classical methods^18^ and deep learning methods^22–24^.

One class of object detection algorithms consists of separate region proposal and classifier networks, such as Fast R-CNN^25^ and Faster R-CNN^26^. However, these networks tend to be slower for real-time applications. Another class of algorithms combines region detection and classification in a single process. Notable examples include Single Shot MultiBox Detector (SSD)^27^ and You Only Look Once (YOLO)^28^. While the R-CNN family achieves high accuracy in detection and classification, recent advancements in the YOLO method have demonstrated superior results in accuracy and speed. However, most object detection algorithms suffer from low sensitivity and may overlook certain objects. This is primarily attributed to being trained on limited datasets that fail to cover all possible scenarios. Consequently, these models struggle to generalize effectively to new images. A recent study by Redmon et al.^29^ investigated the issue of low sensitivity in object detection algorithms. The authors found that these algorithms were prone to missing small objects, partially occluded objects, and objects within cluttered scenes. The paper proposed several methods to enhance the sensitivity of object detection algorithms, including utilizing larger datasets, employing more powerful models, and applying data augmentation techniques. Despite the progress made, further research is necessary to improve the sensitivity of object detection algorithms.

Therefore, for the application of the developed AI model in the mass screening of breast cancer using mammography, we propose an ensemble approach that combines the accuracy of an abnormality classifier model with the explainability of our object detection algorithm. By leveraging both models, we aim to determine the presence of abnormalities in mammograms. The abnormality classifier, trained on a large dataset (as it is less expensive to build compared to the object detection dataset), determines the presence or absence of abnormalities, while the YOLOv5-based detection algorithm locates the abnormality regions within the mammogram.

## Related works

Samuel et al.^18^ used classical machine learning methods for the presence and localization of breast mass. They used pre-processing steps for removing pectoral muscle and other unwanted parts^30^ and used the k-means clustering algorithm to extract a region of interest (ROI), and used classical feature extraction to classify the ROI using support vector machines (SVM). They achieved relatively good performance, but it is limited to mass detection.

Yu et al.^31^ used a modified version of VGG16, which concatenates the feature from each convolutional block using the global average pooling method onto the last flatten layer, to classify patches generated after image processing steps from the MIAS dataset^32^. The pre-processing step includes a median filter for blurring the original image followed by the contrast limited adaptive histogram equalization (CLAHE)^33^. They achieved an F1 score of 0.87 using their proposed model. Although they explain how they generated random ROIs for the negative class, they didn’t mention any method for the positive class.

Vaira et al.^22^ use a VGG-like CNN network to classify ROI generated through a region-growing algorithm. Before applying the region-growing algorithm, labels and pectoral muscles (present in the MLO view) were removed using top-hat morphological operation and thresholding techniques. They still need expert annotation to determine the seed point for the region-growing algorithm to segment the ROI.

Alloqmanin et al.^34^ proposes a pre-trained MobileNetV2 model for feature extraction from and a single-layer perceptron to classify mammography images as normal or abnormal. The framework is evaluated on two public datasets: INbreast and MIAS, and achieves a high AUC-ROC score of 89.79% on the INbreast dataset. The paper also demonstrates the effect of data pre-processing steps on the results and compares the framework with recent and relevant works. The paper claims that the proposed framework overcomes the limitations of previous works and contributes to the development of scientific research in the field of anomaly detection techniques for breast cancer.

Shen et al.^35^ proposed a lightweight deep learning anomaly detection framework for breast cancer diagnosis. The proposed framework uses a pre-trained MobileNetV2 feature extractor and a single-layer perceptron to classify mammography images as benign or malignant. The authors evaluated the framework on two datasets; INbreast and MIAS. To address the problem of data imbalance present in the dataset, they employed Gaussian noise to generate fake data samples.

Lilei et al.^36^ used template matching to extract a region of interest from mammography images and a CNN classifier to classify ROIs into a mass or not. They used a pre-processing step of erosion with $7\times 7$ kernel size followed by dilation with $50\times 50$ kernel size. They prepared a breast mass template and did a template matching across all image regions and selected regions with the highest match. Then, a CNN model was trained and used to extract regions with mass. Finally, they used particle swarm optimization (PSO) to refine the bounding box. Their detection algorithm achieved a fair F1 score of 66.31 using the DDSM dataset.

Mugahed et al.^37^ trained the YOLO9000^38^ algorithm on detecting breast lesions using the INbreast^39^ and DDSM datasets^40^. After the detection, they compared three different classifiers to classify them into benign and malignant. The trained YOLO detector achieved F1 scores of 99.28% and 98.02% for DDSM and INbreast datasets respectively. Of the three classifiers they compared, the InceptionResNetV2 classifier achieved the highest accuracy at 97.5%. However, the researchers have selectively sampled the DDSM dataset for training and testing, which has limited the overall understanding of the model’s performance.

In their research, Ghada et al.^23^ compared YOLO (You Only Look Once) versions 1, 2, and 3 to detect breast masses using the INbreast dataset. They investigated different input image sizes (448, 608, and 832) and anchor numbers (6, 9, and 12) specifically for YOLO v3. The best performance, with a mean Average Precision (mAP) of 77.8 at 0.5 Intersection over Union (IoU), was achieved using an image size of $832\times 832$ and 12 anchors generated through the k-means algorithm.

Hwejin et al.^41^ use a RetinaNet-based breast mass detection model and evaluate its performance on both public and in-house datasets. The model achieved a false positive rate of 0.34 for model confidence as high as 0.95. The authors describe several clinical application challenges, including addressing mass malignancy determination and overcoming data shortage issues. The authors emphasize the need for larger training sets and diverse cases to improve the model’s generalization capabilities.

**Figure 1:**
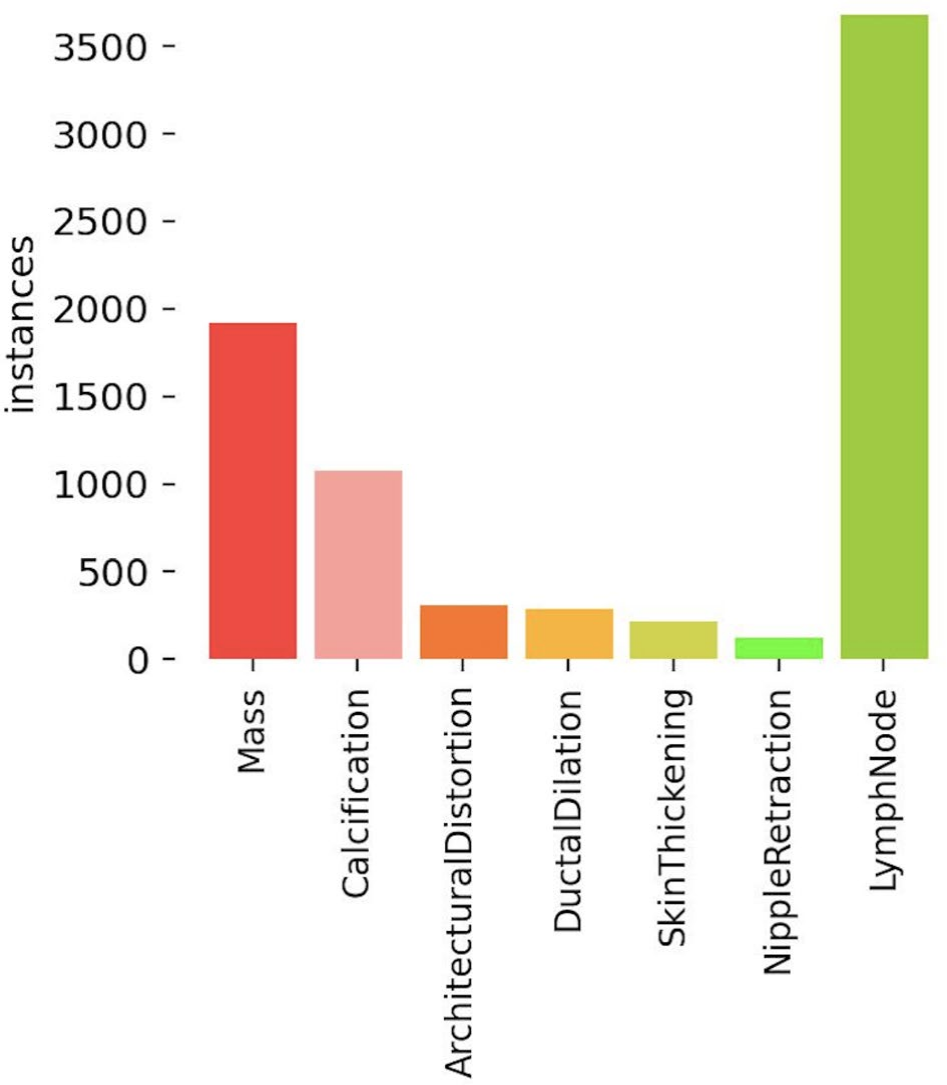
Dataset distribution.

## Ethical approval

Our research was conducted following the ethical principles set forth by the National Ethical Review Committee (NERB). The Addis Ababa Health Bureau institutional review board Committee approved our study and provided us with an official letter reference number of *AAHB*/16591/227. We obtained informed consent from all adult female patients who underwent breast imaging.

## Dataset

In this paper, a dataset for breast-wise abnormality classification was constructed using two primary sources. The first source is VinDr-mammo^42^, which consists of approximately 5000 studies of four-view mammography exams. This dataset includes breast-level assessment and finding annotations. The second source is a locally prepared dataset comprising 3123 breast scans obtained from 1028 patients. Lastly we used the Mini-DDSM^43^ dataset for the purpose of model evaluation. The Mini-DDSM dataset contains 679 CC and MLO scanned breast mammography views belonging to 679 unique cancer cases. The dataset also contains 2408 images of 602 unique patients with normal mammography readings.

To ensure the reliability of the local dataset, annotations were independently performed by two different radiologists. In cases where minor disputes arose, a jury was involved to resolve them. The dataset encompasses annotations for seven types of common breast abnormalities typically reported in mammography screening. These abnormalities include masses, calcification, architectural distortion, lymph nodes, skin thickening, nipple retraction, and asymmetry Fig. 1. We have not used asymmetry since we should consider all four views, which will diminish the size of the dataset though the data has an annotation for breast asymmetry. Additionally, the dataset includes breast-wise BI-RADS level classification.

For training our object detection algorithms to support the decision-making process of the classifier, we filtered scans that contained annotations for lymph nodes, masses, architectural distortions, calcification (specifically Birads 3 and above, as it tends to produce false positives), skin thickening, and nipple retraction.

Due to the limited availability of annotations for abnormalities in our local dataset, we combined annotations from the VinDr-mammo dataset, which specifically covered masses with BI-RADS 3 and higher. By merging these datasets, we generated a suspicious mass dataset comprising 3800 annotations for masses with BI-RADS 3 and above. This focus on the masses is justified as they are the most likely indicators of breast cancer^44^.

During our examination of the VinDr-mammo Dataset^42^ annotations, we identified certain inconsistencies compared to our local dataset. Some abnormalities like calcifications and lymph nodes were labeled as normal in the VinDr-mammo dataset but were identified as abnormalities in our collected dataset. As a result, we decided to rely solely on the suspicious mass detection model to reinforce the decision-making process for abnormality classification. Hence, we utilized the abnormality detection model to offer further insight into the model’s decision (refer to section “Methodology”) and prioritize patient admission.

This exclusive approach was necessary because including the abnormality detection model for determining abnormality led to a significant number of false positives. The main reason for this issue lies in the differences in data annotation between the two datasets.

## Methodology

**Figure 2:**
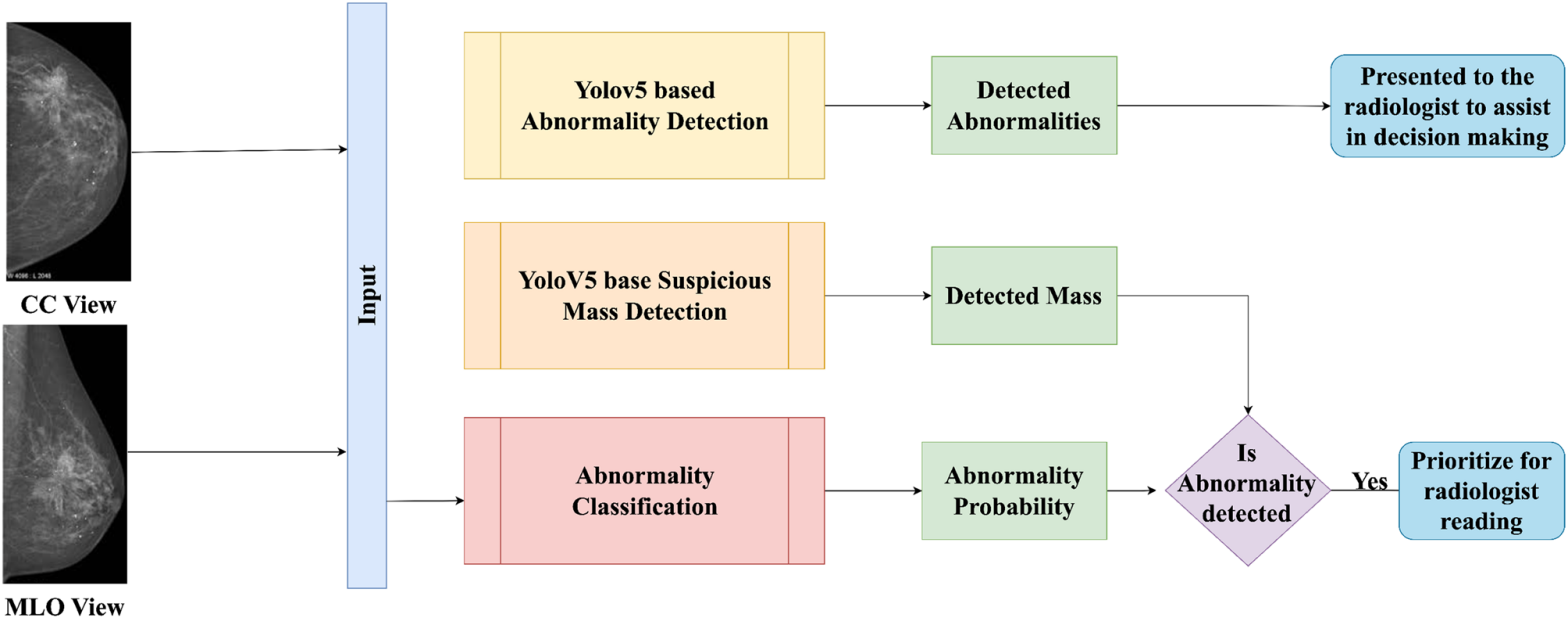
Overall approach.

All research methods described in this study were performed in strict accordance with the ethical principles set forth by the National Ethical Review Committee (NERB). The study protocol was approved by the Addis Ababa Health Bureau institutional review board Committee (Reference Number: AAHB/16591/227). Informed consent was obtained from all adult female patients who underwent breast imaging, and their confidentiality and privacy were rigorously maintained throughout the study.

**Figure 3:**
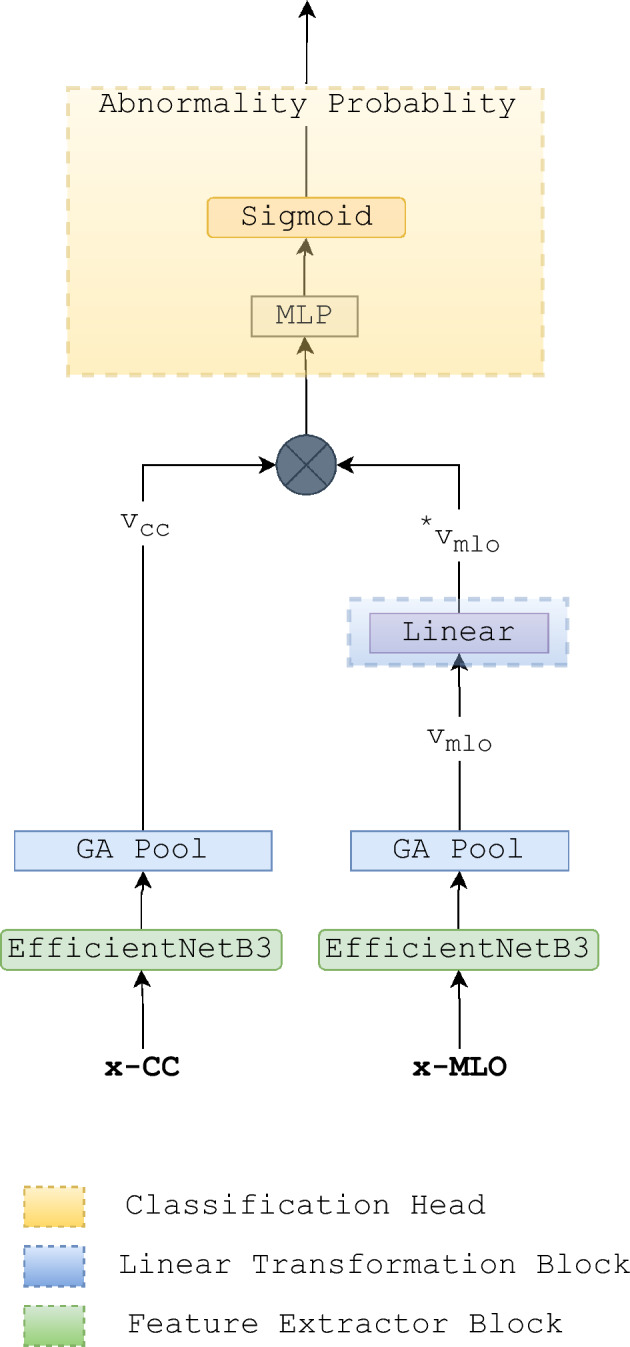
Breast-wise abnormality detection architecture.

We propose a system that utilizes an ensemble of different YOLO models for object detection, along with an EfficientNetB3-based classifier (Fig. 3). This system aims to detect breast abnormalities by considering both CC and MLO views. The first YOLOv5 model is trained specifically to detect suspicious masses from a single mammography image, while the second YOLOv5 model is designed to detect a wide range of abnormalities requiring further screening, ranging from BI-RADS2 to BI-RADS 5. Our third model is an EfficientNetB3-based abnormality classifier, which predicts the probability of abnormality in a breast by simultaneously considering both the CC and MLO views.

We employ object detection models for two main purposes. Firstly, they help explain the decisions made by the classifier, providing insights into the reasoning behind its predictions. Additionally, they assist the classifier in not overlooking any suspicious masses, thereby reducing false positives and improving the overall screening process. The abnormality detection model is utilized to aid radiologists in decision-making and to prioritize examinations, particularly when screening a large number of normal cases.

The overall methodology we adopted for this paper can be generalized in Fig. 2. To determine which examinations should proceed for further screening, we follow the steps outlined below:

- **Step 1:** Acquire the CC and MLO views of the breast, either left or right.
- **Step 2:** Perform breast mass object detection on both breast views.
- **Step 3:** Perform object detection of common breast abnormalities on both breast views.
- **Step 4:** Estimate the probability of breast abnormality by considering both views.
- **Step 5:** If the estimated breast-wise abnormality probability exceeds a threshold of 0.5 and the objectness score for suspicious mass detection is 0.25 or higher, proceed to the next step.
- **Step 6:** Sort patients based on the decision, with those having detected suspicious masses screened first by the radiologist, followed by patients identified as abnormal by the classifier, patients with detected abnormalities by the abnormality detector, and lastly, patients identified as normal by the system.

### Breast-wise abnormality classification

To classify breast images as either normal or abnormal, we use an architecture that accepts both CC and MLO views, passes each view through an EfficientNet-B3 feature extractor separately, and then transforms and concatenates the output feature vectors linearly to produce the input feature vector for the classification head. Our approach utilizes both cranio-caudal (CC) and mediolateral oblique (MLO) views, employing separate instances of the EfficientNet-B3 feature extractor. By applying linear transformations and concatenating the resultant feature vectors, we achieve a consistent input representation for the classification head. To ensure uniformity, we horizontally mirror breast views exhibiting right laterality. Our architecture comprises three key components: the feature extraction block, the linear transformation block, and the classification head Fig. 3. Specifically, the feature extraction block leverages two pre-trained EfficientNet-B3 networks, renowned for their superior accuracy, reduced parameters, and computational efficiency compared to alternative convolutional neural networks (CNNs) like ResNet and Inception. EfficientNet-B3 adopts a compound scaling approach, scaling depth, width, and resolution simultaneously. With 26 layers and approximately 12.2 million parameters, it is designed to optimize efficiency while maintaining accuracy. Notably, the architecture of EfficientNet-B3 encompasses a stem convolutional layer, a series of repeating blocks integrating depthwise and pointwise convolutions, squeeze-and-excitation (SE) blocks, and skip connections. For our framework, we employ the ImageNet weights for the top CNN blocks of EfficientNet-B3 and fine-tune the weights on the upper layers using our dataset. Additionally, the linear transformation block, comprising a single linear block, receives the MLO view feature vectors obtained from the EfficientNet-B3 feature extraction block as input. The primary objective of the linear transformation block is to learn a linear mapping from the MLO view to the CC view, as expressed in Eq. (1).

1 $$\begin{aligned} v^{*}_{mlo} = w^{T}v_{mlo} \end{aligned}$$

where $v^{*}_{mlo}$ is the transformed feature vector $v_{mlo}$ with learnable parameters *w*.

The classification head is the concatenation of the feature extractor output for the CC view and the linear transformation block output of the MLO view. The architecture was trained to optimize a loss function *L* Eq. (2), which is formulated from the combination of the cross-entropy loss of the classification head Eq. (3), and cosine similarity loss of the linear transformation block Eq. (4).

2 $$\begin{aligned} L = \alpha [CE_{loss}] + \beta [\cos (\theta )_{loss}] \end{aligned}$$

Where,

3 $$\begin{aligned} CE_{loss} = -\frac{1}{N}\sum _{i=1}^{N} [y_i \log {\bar{y_i}} + (1-y_i)\log (1-\bar{y_i})] \end{aligned}$$

4 $$\begin{aligned} \cos (\theta )_{loss} = 1 - \frac{v_{cc}^T v^{*}_{mlo}}{||v_{cc}|| ||v^{*}_{mlo}||} \end{aligned}$$

where $\mathbf {v_1}$ and $\mathbf {v_2}$ are two non-zero feature vectors of the two breast views. For, $0 \le \alpha , \beta \le 1$ To train the breast-wise abnormality detection model, we used a total of 26,056 mammography images belonging to 6514 studies. The datasets are divided into groups of studies, where each unique study contains four mammography images and two images for each breast laterality. We reserved 20% of the data as a test set for the cross validation. We performed a 5-fold group cross-validation, where samples are grouped based on their study IDs. We used AUC-ROC, f1-score, precision, recall, and accuracy metrics for model evaluation. To fortify the robustness of our prediction models, we meticulously conducted cross-validation with a comprehensive approach. The dataset was initially partitioned into training and testing sets using a fixed random seed, ensuring the reproducibility of our results. Subsequently, a rigorous 5-fold cross-validation procedure was employed on the training set, wherein the entire model development pipeline was iteratively executed during each fold. It is crucial to underscore that at the culmination of each fold, the model underwent evaluation on the designated test set, which had been carefully excluded from any aspect of the model development process. For abnormality detection, we grouped each study according to their BI-RADS category. BI-RADS is a standardized system used by radiologists to describe the results of breast imaging tests. The BI-RADS system assigns a category to each breast imaging study, ranging from 1 (negative) to 6 (known biopsy-proven malignancy) Table 1.

- **Category 1: Negative** No abnormal findings
- **Category 2: Benign** There is a very low probability of cancer.
- **Category 3: Probably benign** There is a slightly increased probability of cancer.
- **Category 4: Suspicious for malignancy** There is a moderate to high probability of cancer.
- **Category 5: Highly suggestive of malignancy** There is a very high probability of cancer.
- **Category 6: Known biopsy-proven malignancy.** Cancer has been confirmed by biopsy.

**Table 1:** BI-RADS distribution of the used dataset.

Category	Number of studies
BI-RADS 1	15,773
BI-RADS 2	7039
BI-RADS 3	1574
BI-RADS 4	1304
BI-RADS 5	366

For the task of abnormality detection, we classified studies with BI-RADS of category 2 and above as ’Abnormal’ and studies with BI-RADS category 1 as ’Normal’. Overall we trained the architecture on Nvidia Tesla T4 GPU for a maximum of 100 epochs with early stopping. We used the Adam optimizer and we experimented with various learning rates of $1 \times 10^{-1}$, $1 \times 10^{-2}$, $1 \times 10^{-3}$, and $3 \times 10^{-4}$. We found that a learning rate of $3 \times 10^{-4}$ is the best learning rate in terms of mean classification accuracy.

### Object detection

We trained YOLOv5 models of various sizes (small, medium, normal, large, and extra large) to detect breast abnormalities in mammographic images. YOLOv5 is a state-of-the-art object detection algorithm based on the You Only Look Once (YOLO) architecture^45^. Introduced in 2020^46^, YOLOv5 is an improved version of its predecessors, YOLOv4 and YOLOv3.

The YOLOv5 architecture consists of three main components: a backbone network, a neck network, and a head network. The backbone network, a convolutional neural network (CNN), extracts features from the input image. The neck network combines features from different scales, while the head network predicts bounding boxes and class probabilities for objects in the image. YOLOv5 employs an anchor-based approach for object detection, where each anchor box is associated with a specific aspect ratio and scale. The anchor boxes are used to predict object bounding boxes and class probabilities are determined using a softmax function.

One notable feature of YOLOv5 is its use of a Siamese network, enabling parallel image processing. This allows YOLOv5 to maintain high accuracy while processing images faster than its predecessors. YOLOv5 has demonstrated state-of-the-art performance on various benchmarks, including the COCO dataset^47^.

In addition to its object detection capabilities, the YOLOv5 models contribute to the explainability and interpretability of the classifier system. By detecting and localizing breast abnormalities, the models provide visual evidence for the classification decisions. This interpretable nature of the system enhances transparency and allows medical professionals to understand the basis of the classifier’s predictions. Consequently, the object detection algorithms not only improve the sensitivity of the overall system but also provide insights into the diagnostic process through explainable results.

To ensure accurate object detection, we ensemble the predictions of the trained models and apply Non-Maximum Suppression (NMS). Utilizing object detection models with explain-ability/interpret-ability features enhances the sensitivity and transparency of the overall system, particularly for screening purposes in breast cancer detection.

We have trained the Yolov5 models for two sets of tasks, Suspicious mass detection and Other Abnormality detection. We used the suspicious mass detection model to assist the normal abnormal classifier in the abnormality detection. We also trained the other abnormalities classifier for the detection of abnormalities, not only mass but also lymph nodes, calcifications, architectural distortions, ductal dilation, and nipple retraction.

## Results

### Classification model

**Figure 4:**
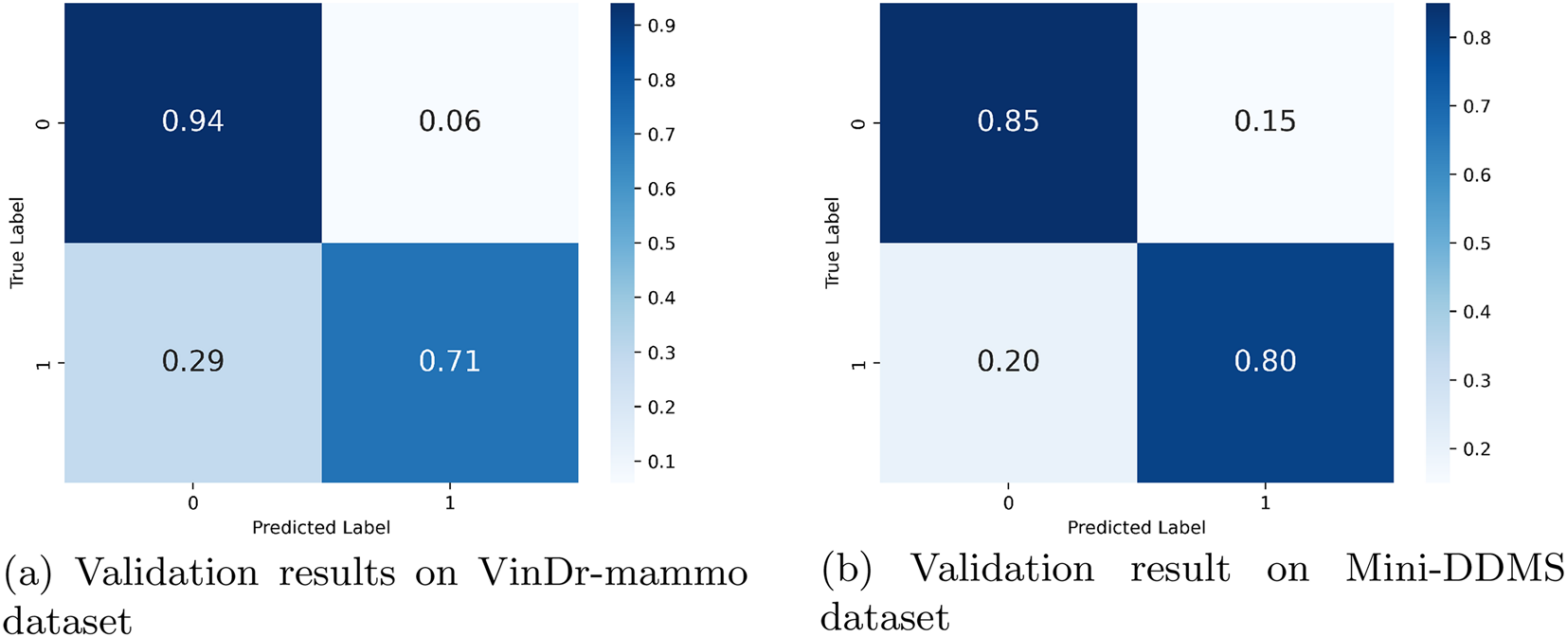
Confusion matrix of the 5-fold cross validation results.

**Figure 5:**
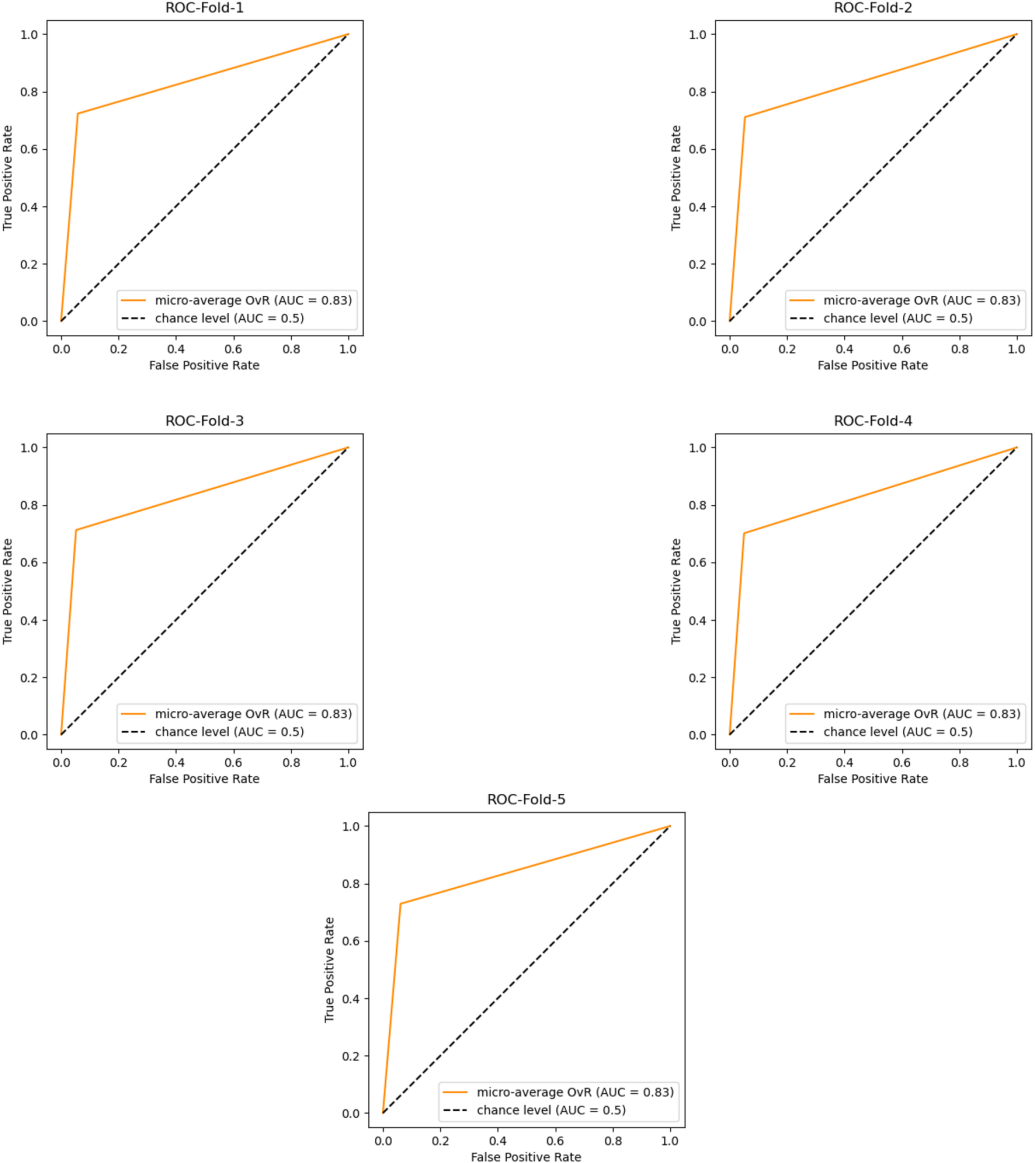
Receiver operating characteristic (ROC) curves and area under the curve (AUC) values for a 5-fold cross-validation of the breast-wise abnormality classifier.

We performed testing at the end of each fold and we calculated the AUC-ROC and the Confusion matrix. We evaluated the models on two test sets; Hold out test data from the VinDr-mammo dataset and on the Mini-DDMS dataset. On the VinDr-mammo dataset, the breast-wise abnormality classifier achieved a mean cross-validation precision, recall, f1 score, and AUC of 0.91, 0.82, 0.87 and 0.83 respectively Table 2. On the Mini-DDMS dataset, the model achieved a mean precision, recall, f1 score, and AUC of 0.82, 0.82,0.82, and 0.82 respectively. Confusion matrix for each 5-fold validation is given in Figs. 4, 5.

**Table 2:** Performance comparison of the breast-wise abnormality detection network and ensemble on five-fold cross-validation on VinDr-mammo dataset.

	Breast-wise			Ensemble		
	Precision	Recall	F1	Precision	Recall	F1
Mean	0.91	0.82	0.87	0.71	**0.89**	0.79

Significant value is in bold.

### Object detection models

**Figure 6:**
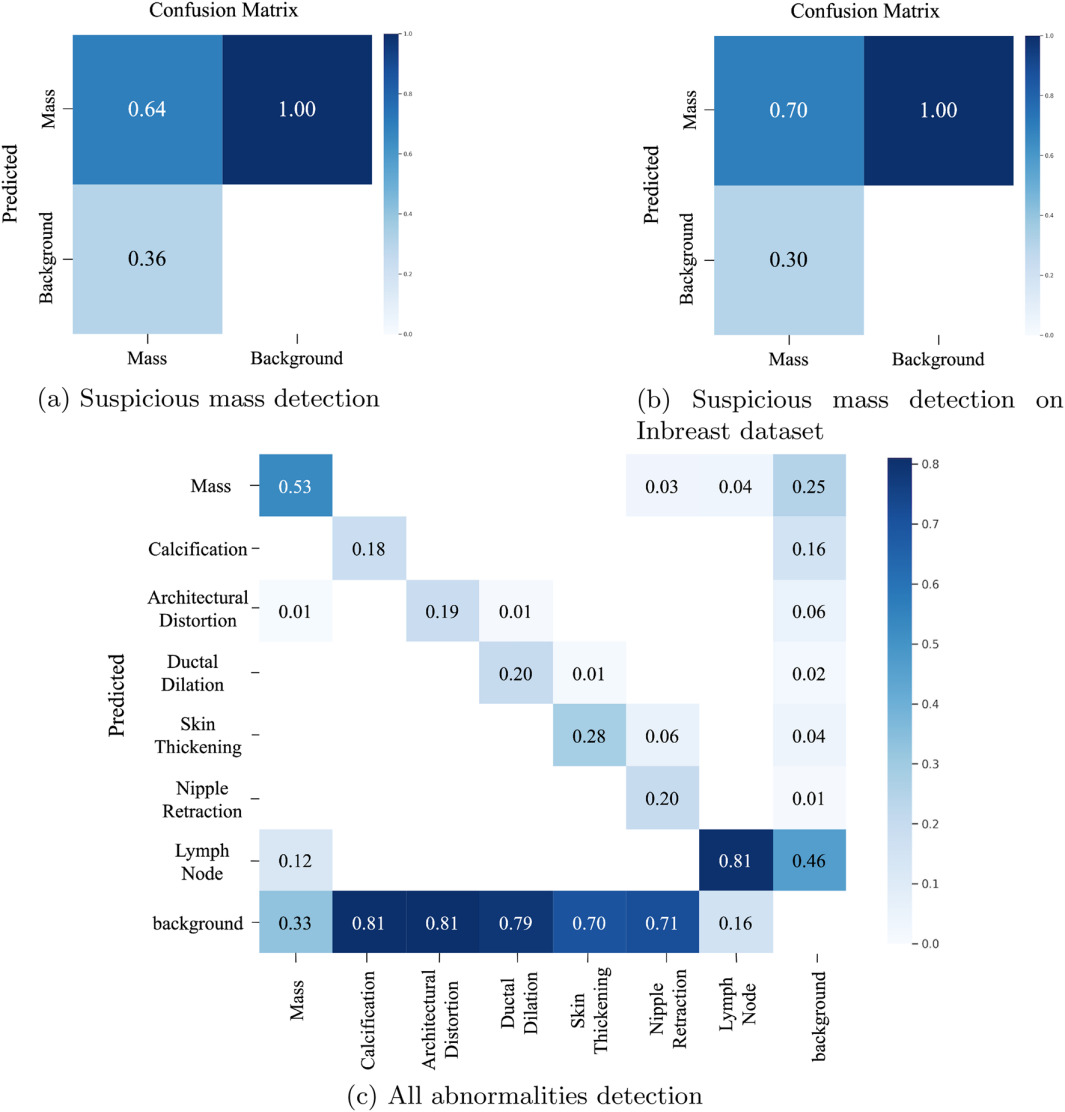
Confusion matrix: Yolo models.

**Figure 7:**
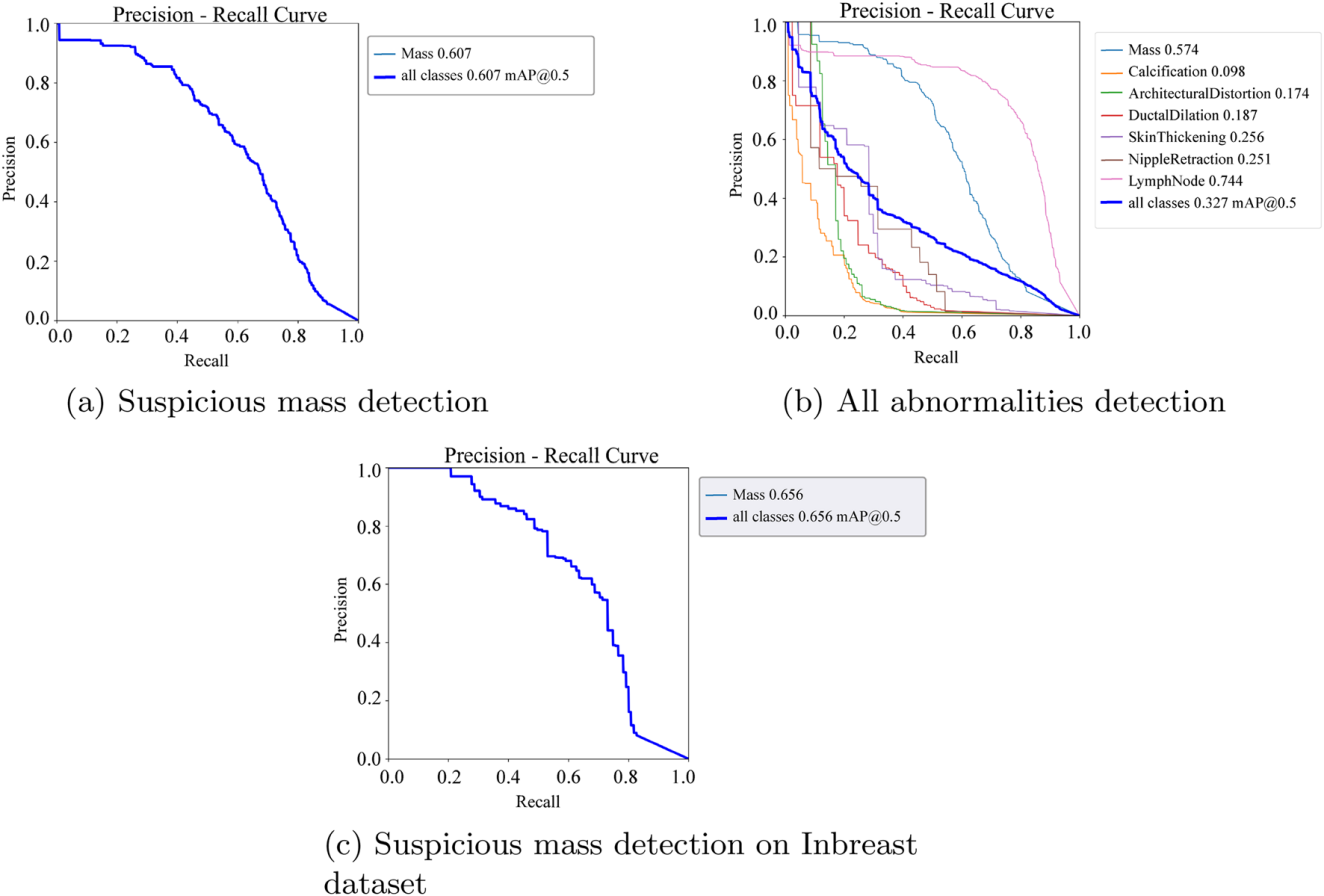
PR curve: Yolo models.

We trained and evaluated the suspicious mass detection models and the other abnormality detection models separately. The addition of VinDr-mammo’s suspicious mass annotations significantly improved mass detection performance, as demonstrated in Figs. 6 and 7. When trained solely on the local dataset, the object detection algorithm achieved an mAP of 0.57. However, with the inclusion of VinDr-mammo’s annotations, the mAP improved to 0.67.

The proposed suspicious mass detection model demonstrated similar performance on the Inbreast dataset. The incorporation of VinDr-mammo’s mass annotations enhanced the robustness of the suspicious mass detection, as evidenced by its performance on the VinDr-mammo dataset.

It is important to note that the performance of the object detection models decreased for most classes, except for mass and lymph nodes. This decline in performance can be attributed to the low representation of these classes in the training dataset. Although we had sufficient representation for calcifications in the dataset, the detection performance was poor due to the small size of these annotations, which makes them more challenging to train and detect using YOLO models.

In the confusion matrix plots, it is important to note that there are no true background predictions because the model only outputs predictions for the mass class and not for the background class. Therefore, it is more appropriate to consider only the outputs of the positive class when evaluating the model’s performance. In such cases, average precision is a suitable metric to assess performance in most object detection algorithms.

The abnormality classifier trained on the local dataset performs well in detecting lymph nodes. This is likely because lymph nodes are well-represented in the dataset and are relatively easy to detect. The increased representation of lymph nodes in the training data allows the model to learn more effectively and achieve better results for this class.

Figure 6 shows that the majority of mislabeling occurs between the mass and lymph node classes. This is consistent with the challenges that radiologists face in distinguishing between these two classes in some cases. The similarities in appearance and characteristics between masses and lymph nodes can lead to mis-classifications.

The performance of the lymph node class, with an mAP of 0.74, and the observation that increased representation improves performance, further support the importance of having a sufficient number of samples for each class in the dataset. The more representative instances there are during training, the better the model’s ability to accurately detect and classify different abnormalities.

### Ensemble model

For mass screening of breast cancer cases, the most important factor we need to track is the sensitivity of the network, which implies the percentage of actual true cancer cases detected. We can improve the sensitivity(recall) of the network by adopting the abnormality detection presented in Fig. 2, where we ensembled the predictions of the YOLO mass detection network and the breast-wise abnormality network. To evaluate the ensembled network we selected a subset of the VinDr-mammo test set we used for evaluating the breast-wise abnormality detection network but excluded studies with no corresponding bounding box information. As we can see from Table 2, ensembling the results of the two networks, we can improve the sensitivity by a good margin. This is an important factor in designing a mass screening tool, where we need the highest possible detection rate of actual suspicious cases.

**Figure 8:**
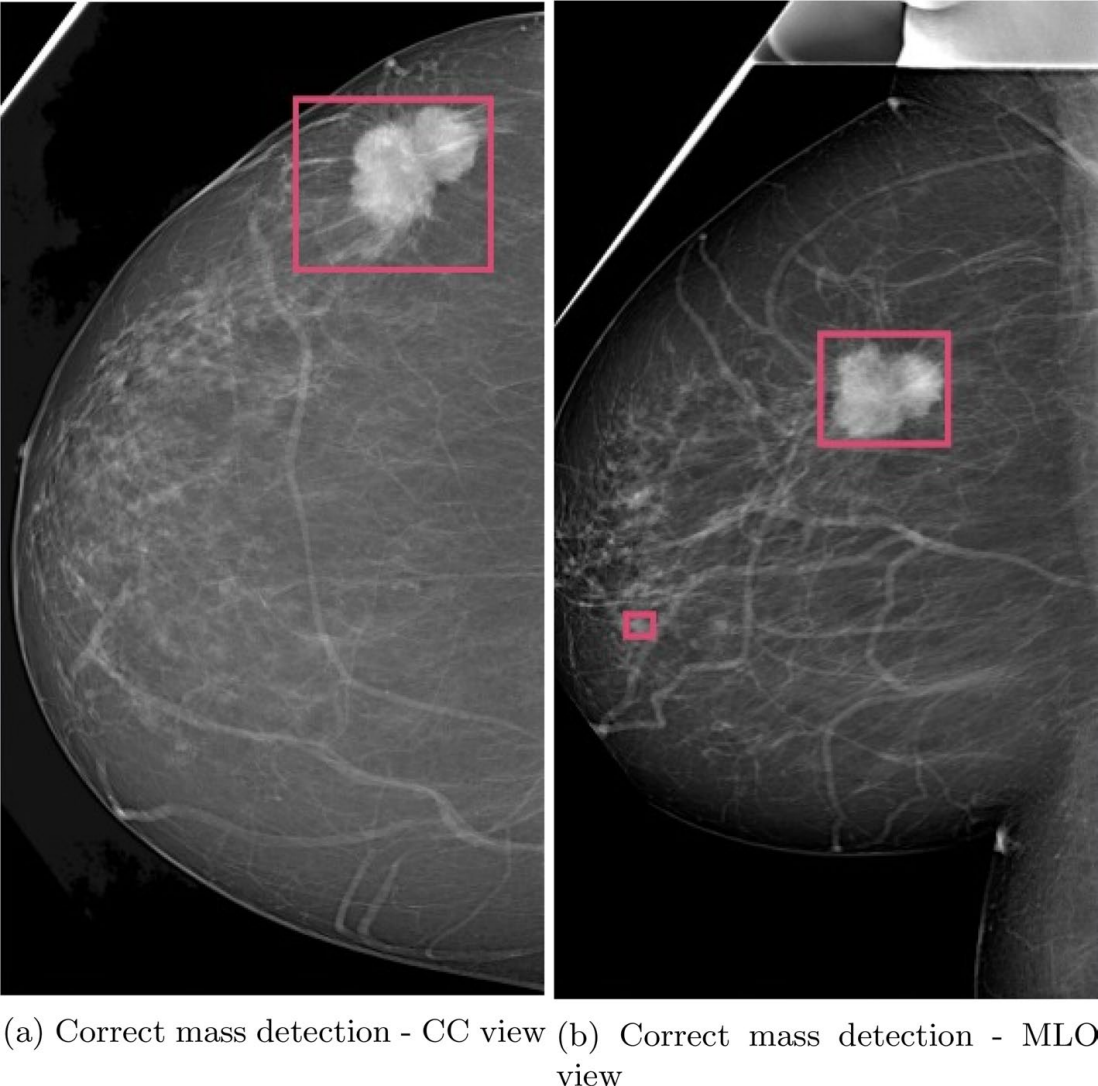
Object detection model supporting correct classifier decisions for mass detection.

**Figure 9:**
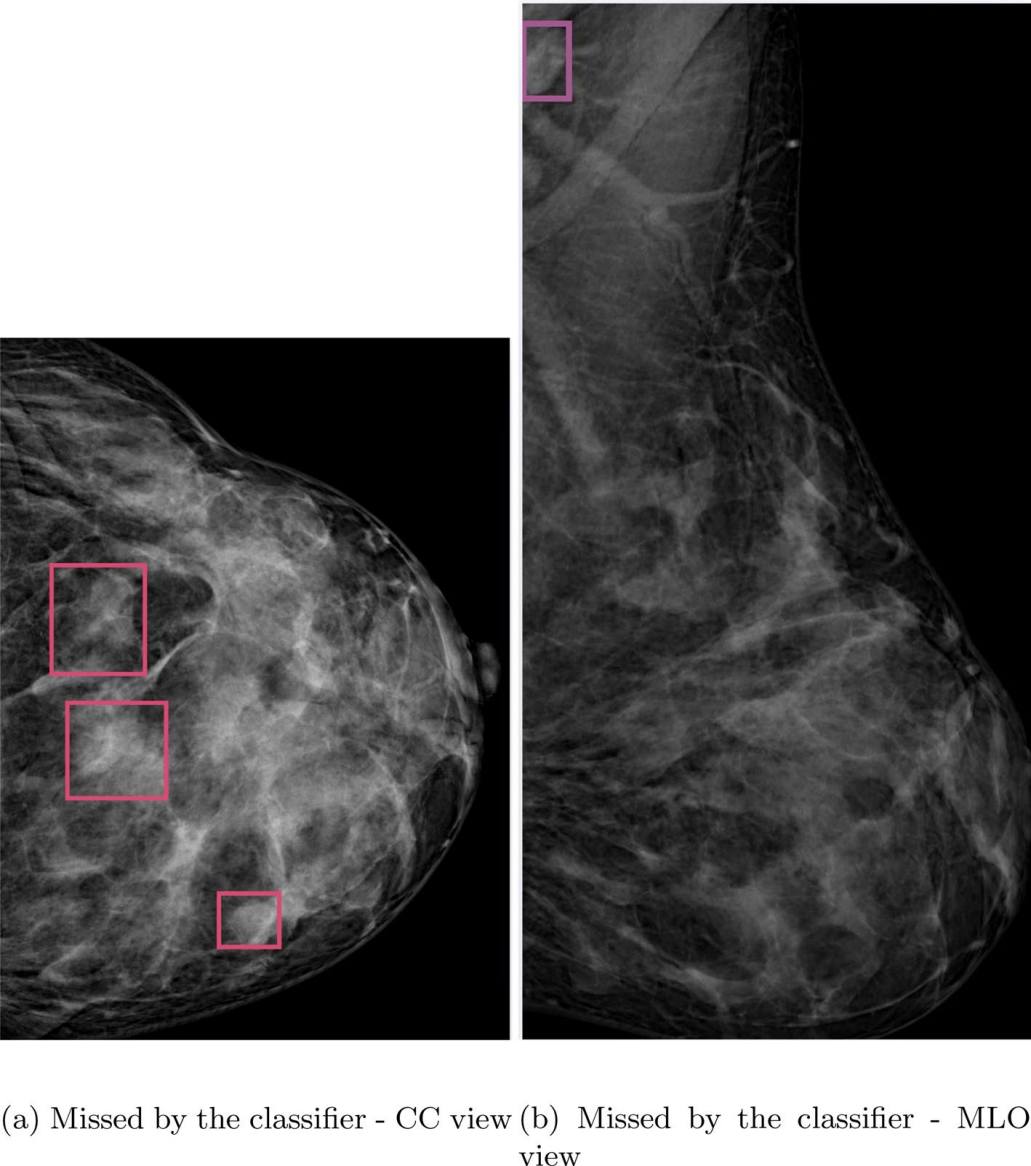
Object detection model supporting classifier models to avoid missing masses.

**Figure 10:**
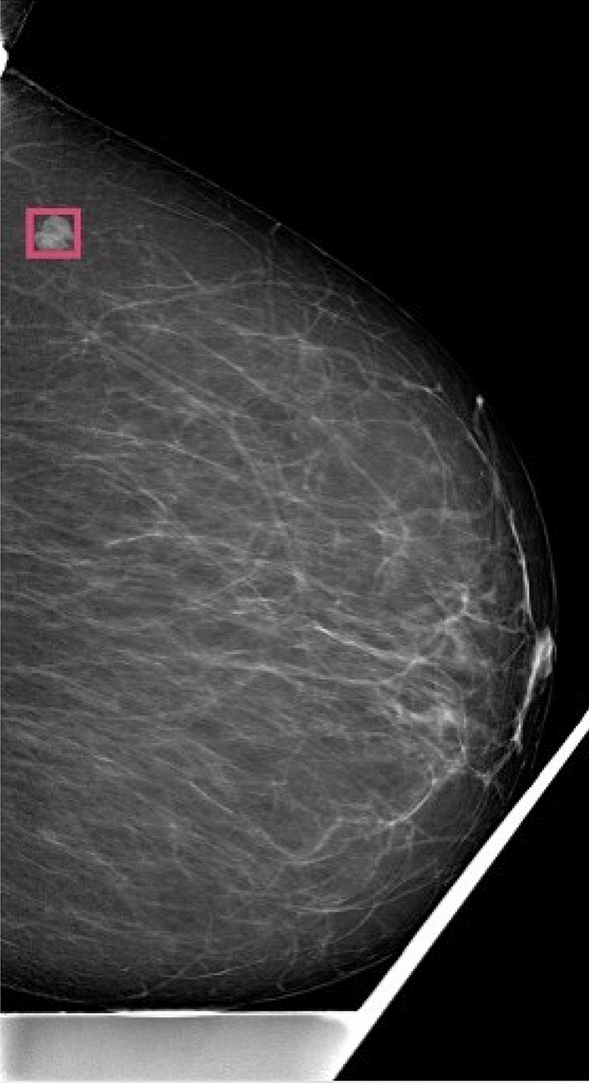
A lymph node was incorrectly labeled as suspicious mass.

Figure 8 shows that the suspicious mass detection algorithm is effective in identifying the type and location of abnormalities. This enhances the interpretability of the classifier model’s decision, making it easier for radiologists to understand. Additionally, using the detection of suspicious masses as an indicator of abnormality increases the system’s sensitivity. Figure 9 shows that the object detection model can still identify masses, even if the classifier misses the abnormality. This further increases the system’s sensitivity in detecting potential abnormalities. However, it is important to note that this increased sensitivity can also introduce false positives, as shown in Fig. 10. This highlights the possibility of the model incorrectly identifying certain instances as masses, resulting in false positive predictions. Nonetheless, the all-abnormality detection model is very good at distinguishing between masses and lymph nodes, and it correctly classified the instance in Fig. 10 as a lymph node. Hence, the radiologist should check all abnormality detection model’s outputs for further investigation before he makes his final decision.

These observations highlight the trade-off between sensitivity and specificity in the system. While increased sensitivity increases the chances of detecting abnormalities, it can also lead to false positives. Achieving the right balance between sensitivity and specificity is crucial for optimizing the performance of the system in clinical applications.

**Figure 11:**
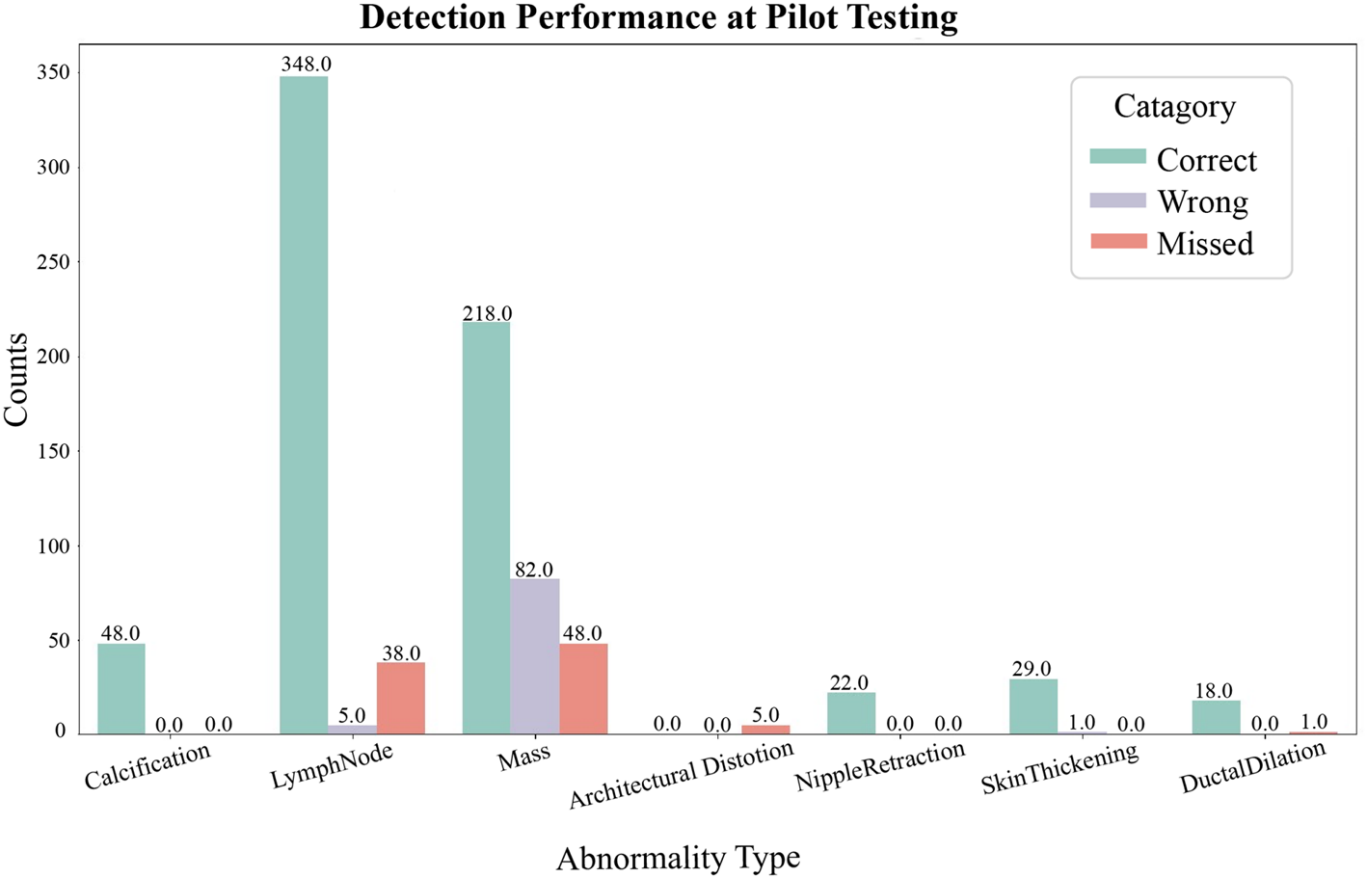
A pilot test result.

We have also conducted real-world testing to validate the performance of the model by involving radiologists. In total, we conducted tests on 360 patients. The overall accuracy of the model in identifying abnormalities was 83.19%. Moreover, the suspicious mass detection model, based on YOLOv5, assisted the abnormality classifier in detecting an additional 85 abnormalities that would have otherwise been missed. Figure 11 demonstrates the sustained testing performance of the suspicious mass detection model, successfully detecting 82% of the masses. However, there were approximately 82 false positives. The best performance was observed in lymph node detection, achieving an accuracy of 89% and correctly identifying 90% of them. On the other hand, the model performed poorly in detecting the architectural distortion abnormality, missing all instances. This can be attributed to insufficient representation in the training dataset and the inherent difficulty of detecting such abnormalities. Regarding calcification, there were no missed or incorrectly annotated cases, as the radiologists only considered calcifications with BI-RADS 3 or above. The performance of other classes was generally very good, but their infrequent occurrence may have contributed to their high performance.

## Conclusion

This paper introduces a method for the detection of breast abnormality for mass screening programs using mammography. Our approach utilizes an ensemble of networks, including an EfficientNet-based breast-wise abnormality detection network that predicts the probability of breast abnormality using CC and MLO views, along with a YOLO suspicious mass detection network. By combining these networks, our method aims to enhance the robustness and sensitivity of the mass screening process, particularly for suspected cancer cases, while also improving the explainability of the AI model.

To evaluate the effectiveness of our method, we conducted experiments on the VinDr-mammo dataset using a 5-fold cross-validation technique. We assessed the performance using precision (specificity), recall (sensitivity), and f1-score metrics. The results demonstrate that our ensemble approach significantly improves sensitivity by 8%. The mean cross-validation precision, recall, and f1-score were found to be 0.71, 0.89, and 0.79, respectively.

Furthermore, the pilot testing results indicate a consistent performance of our approach, similar to what was observed in the VinDr-mammo dataset. However, there are some challenges with abnormalities that have a smaller representation in the training set. To address this, we recommend enhancing the system’s performance by incorporating more data into the training set and improving the classifier model by including different types of datasets to increase its robustness. Additionally, it is advisable to separate the detection of calcifications from other abnormality detection, as their size warrants separate treatment.

## Data Availability

The local data that support the findings of this study are available from the Ethiopian Artificial Intelligence Institute. Still, restrictions apply to the availability of these data, which were used under license for the current study, and so are not publicly available. Data are however available from the authors upon reasonable request and with permission of the Ethiopian Artificial Intelligence Institute. Hence, anyone who wants to have access to the data can contact the corresponding author or directly via the institute’s email (mailto:contact@aii.et).
